# Supplementary material for: Analytical framework for evaluating NMPC-based robot navigation in fluid environments
Source: PLoS One. 2026 Mar 30;21(3):e0333056. doi: 10.1371/journal.pone.0333056 (PMC13035167; doi:10.1371/journal.pone.0333056)
Supplement: Attachment — Submitted filename: Reply to Comments.docx [file pone.0333056.s002.docx]

**Original Manuscript ID:** **PONE-D-25-48180R1**

Original Article Title: “Analytical Framework for Evaluating NMPC-Based Robot Navigation in Fluid Environments’’

**To:** PLOS ONE Editor

**Re:** Response to Editor

Dear Editor,

Thank you for allowing a revision of our manuscript, with an opportunity to address the Editor’s comments.

We are uploading (a) our point-by-point response to the comments (below) (response to Editor, under “Response to Editor*”*), (b) an updated manuscript with blue text indicating changes (as “Revised Manuscript with Track Changes*”*), and (c) a clean updated manuscript without highlights (“Manuscript”*).*

Best regards,

Maram Ali, Saptarshi Das and Stuart Townley

**Editor’s Comment** **#1**

**Editor’s Comment #1, Concern #1:**

The authors are suggested to perform grid independence analysis and include results of grid independence in the revised paper.

**Author Response**:

We thank the Editor for this constructive suggestion. We agree that a robust grid independence study is essential to validate the numerical reliability of the Fluid-Structure Interaction (FSI) results used within our NMPC framework. We have conducted a systematic grid sensitivity analysis using four lattice resolutions (10 ×10, 50× 50, 100 ×100, and 200 ×200) to evaluate the trade-off between numerical accuracy and computational overhead.

**Author Action:**

We have added a new subsection titled "Numerical Validation and Grid Convergence" in the revised manuscript as section 3.2. This section includes:

1. Table 2, which provides a quantitative comparison of relative force error, vorticity fidelity, runtime, and memory allocation across the tested resolutions.
2. Figure [1] shows a convergence plot demonstrating the asymptotic decay of the drag force error.
3. An expanded discussion justifying the selection of the 200 × 200 grid as the benchmark resolution that ensures a grid-independent and physically consistent environment for the NMPC evaluations.
